# Curcumin and Photobiomodulation in Chronic Viral Hepatitis and Hepatocellular Carcinoma

**DOI:** 10.3390/ijms21197150

**Published:** 2020-09-28

**Authors:** Laura Marinela Ailioaie, Gerhard Litscher

**Affiliations:** 1 Department of Medical Physics, Alexandru Ioan Cuza University, 11 Carol I Boulevard, 700506 Iasi, Romania; lauraailioaie@yahoo.com; 2 Ultramedical & Laser Clinic, 83 Arcu Street, 700135 Iasi, Romania; 3 Research Unit of Biomedical Engineering in Anesthesia and Intensive Care Medicine, Research Unit for Complementary and Integrative Laser Medicine, and Traditional Chinese Medicine (TCM) Research Center Graz, Medical University of Graz, Auenbruggerplatz 39, 8036 Graz, Austria

**Keywords:** blue laser, hepatitis B, hepatocellular carcinoma, laser blood irradiation, photodynamic therapy, ultrabioavailability

## Abstract

Immune modulation is a very modern medical field for targeting viral infections. In the race to develop the best immune modulator against viruses, curcumin, as a natural product, is inexpensive, without side effects, and can stimulate very well certain areas of the human immune system. As a bright yellow component of turmeric spice, curcumin has been the subject of thousands of scientific and clinical studies in recent decades to prove its powerful antioxidant properties and anticancer effects. Curcumin has been shown to influence inter- and intracellular signaling pathways, with direct effects on gene expression of the antioxidant proteins and those that regulate the immunity. Experimental studies have shown that curcumin modulates several enzyme systems, reduces nitrosative stress, increases the antioxidant capacity, and decreases the lipid peroxidation, protecting against fatty liver pathogenesis and fibrotic changes. Hepatitis B virus (HBV) affects millions of people worldwide, having sometimes a dramatic evolution to chronic aggressive infection, cirrhosis, and hepatocellular carcinoma. All up-to-date treatments are limited, there is still a gap in the scientific knowledge, and a sterilization cure may not yet be possible with the removal of both covalently closed circular DNA (cccDNA) and the embedded HBV DNA. With a maximum light absorption at 420 nm, the cytotoxicity of curcumin as photosensitizer could be expanded by the intravenous blue laser blood irradiation (IVBLBI) or photobiomodulation in patients with chronic hepatitis B infection, Hepatitis B e-antigen (HBeAg)-positive, noncirrhotic, but nonresponsive to classical therapy. Photobiomodulation increases DNA repair by the biosynthesis of complex molecules with antioxidant properties, the outset of repairing enzyme systems and new phospholipids for regenerating the cell membranes. UltraBioavailable Curcumin and blue laser photobiomodulation could suppress the virus and control better the disease by reducing inflammation/fibrosis and stopping the progression of chronic hepatitis, reversing fibrosis, and diminishing the progression of cirrhosis, and decreasing the incidence of hepatocellular carcinoma. Photodynamic therapy with blue light and curcumin opens new avenues for the effective prevention and cure of chronic liver infections and hepatocellular carcinoma. Blue laser light and UltraBioavailable Curcumin could be a new valuable alternative for medical applications in chronic B viral hepatitis and hepatocarcinoma, saving millions of lives.

## 1. Photobiomodulation and Regenerative Medicine

The fascination of regenerative medicine is much stronger today when we are trying more than ever to reverse aging and to trigger the restoration of normal cell, tissue, and organ functions by regeneration, replacement, and repair [1].

Since their invention, low-level lasers (from red to near infrared), or more recently named photobiomodulation, applied in medicine proved to have beneficial effects on relieving pain, decreasing swelling and inflammation, and stimulating the healing of the living tissues, when used in appropriate doses.

More than 50 years after their first medical application, the biologically accepted effects of lasers are on cytochrome c oxidase in the mitochondria, increasing ATP production; modulating reactive oxygen species, nitric oxide, and the calcium ion levels; activating a multitude of transcription factors; improving the cellular metabolism; and increasing the synthesis of new proteins and cell proliferation and migration [2].

Nowadays, lasers and molecular medicine considering tissue repair in infectious diseases and cancers are inventions that gain control of our imagination in the attempt to defy illness and death [1].

Important advances in our understanding of the basic science of lasers’ high-tech applications and photobiomodulation are constantly influencing the development of laser technology and the use of it. Translation of preclinical regenerative medicine into advanced therapy combined with nanotechnologies and natural products will lead to future developments, which protect the patients, reduce healthcare costs. and offer huge potential benefits.

Understanding and explaining all known photobiomodulation effects optimize future methods and extremely increase the effectiveness of low-level laser therapy as a modern approach in the treatment of chronic infections or inflammatory processes.

Numerous associated and combined photobiomodulation techniques were developed and are widely used nowadays: Locally, on the projection of internal organs; laser acupuncture; intracavitary, transdermal, and intravenous laser blood illumination; magnetic-laser therapy; laser phoresis; etc. [3].

## 2. Chronic Hepatitis B Infection

Hepatitis B is a DNA virus that infects liver cells and can cause both acute and chronic disease. Hepatic infection is one of the main causes of cirrhosis, hepatic decompensation, and hepatocellular carcinoma and, even in this third millennium of modern medicine, there are no satisfactory therapies for chronic hepatitis B virus. Both viral and host factors are responsible for determining whether the infection is cleared or becomes chronic. It is estimated that over 250 million people worldwide are positive for the surface antigen of hepatitis B, HBsAg [4].

In May 2016, the World Health Assembly endorsed the Global Health Sector Strategy (GHSS) on viral hepatitis 2016–2021. The GHSS calls for the elimination of viral hepatitis as a public health threat by 2030, reducing new infections by 90% and mortality by 65% [4].

Chronic liver disease is responsible for millions of deaths annually and is characterized by permanent inflammatory processes that predispose to liver cancer and hepatocellular carcinoma. In healthy liver, inflammatory processes stimulate growth and repair and restore normal liver architecture. However, if liver inflammation becomes chronic, the balance of damage versus regeneration in the liver is disrupted and can lead to the formation of excessive scar tissue (fibrosis) [5].

In the long term, an exacerbation of fibrosis will lead to cirrhosis, which is characterized by abnormal liver architecture and function and is associated with a significant reduction in overall health and well-being. At cirrhotic stages, liver damage is often irreversible or difficult to treat. Cirrhosis leads frequently to death from liver failure or to hepatocellular carcinoma (HCC). Indeed, HCC is the first cause of death in cirrhotic patients [6] and is a tumor with poor prognosis, ranking third in terms of death by cancer. Furthermore, it is the fifth most prevalent cancer worldwide, with 800,000 new cases per year in the world [7,8].

In some European countries, like Romania, for example, according with the last studies, the prevalence of chronic hepatitis B (HBV) infection is extremely high. There are almost 1.3 million people with chronic B virus infection and over 600,000 patients are infected with HCV (hepatitis C). Hepatitis C interferon treatment is a past option and the emergence of new drugs opens the perspective of healing without side effects [9].

Currently, there are two therapeutic options for chronic hepatitis B, interferon (IFN) and analog nucleotides (NA). Treatment with interferon should be used with caution in patients with cirrhosis and is contraindicated in case of liver decompensation. Analogous nucleotides are generally well tolerated but have a high risk of developing resistance to them [10].

Therapeutic protocols proposed and used to date have shown that complete elimination of hepatitis B virus (with covalently closed circular DNA (cccDNA)) was not possible due to the resistance to the pharmacological medication used (for example: Lamivudine 70% resistance at five years, Adefovir 30% resistance at five years) [11].

The main objectives of current treatments are suppressing the virus and controlling the disease by: Reducing inflammation/fibrosis and stopping the progression of chronic hepatitis, reversing fibrosis and diminishing the progression of cirrhosis, decreasing the incidence of hepatocellular carcinoma, which is not yet eliminated.

All up-to-date treatments are limited. There is still a gap in the scientific knowledge and a sterilization cure may not yet be possible with the removal of both covalently closed circular DNA (cccDNA) and the embedded HBV DNA.

First theoretical estimations of nucleotide action on blocking the formation of new cccDNA in a patient with chronic hepatitis B predicted that it would take approximately 14 years to eliminate intrahepatic cccDNA. Today we know for sure that nucleotides do not fully stop HBV replication or cccDNA formation [12].

A functional cure is currently achievable in a small percentage (<10%) of patients after IFN or NA therapy. Future strategies should implement new targets and novel immunomodulatory therapies to restore both innate and adaptive immune responses in order to achieve HBsAg loss in a higher percentage (>50%) of patients after completion of a limited course (≥2 years) of treatment. However, special attention should be paid to uncontrolled immune activation that could lead to fatal hepatitis or extrahepatic organ damage [13].

## 3. Antiviral Properties of Curcumin and Applications

Antibacterial, antiviral, and anti-inflammatory properties of curcumin as a highly polyphenolic molecule were discovered many years ago and, since then, its strong antioxidative, antiproliferative, and apoptosis-promoting effects have been studied in many different cells. Safe and well tolerated even at higher doses, curcumin has been used for treating hyperproliferative diseases [14].

Current concerns about hepatitis B therapy include the discovery of new management methods to ensure easy, safe, and low-cost administration for curative and eradicative treatment that effectively and early controls viral replication without developing resistance.

There are studies that have demonstrated the valuable pharmacological impact of curcumin in hepatitis B [15], diabetic microangiopathy, immune-mediated inflammatory diseases, and even cancer [16].

New patented curcumin supplements with a bioavailability of 15,000 times higher offer the chance of increased efficiency on the natural product [17], which is unstable at physiological pH and has a low water solubility and a fast metabolism [16,17].

Experimental studies have highlighted the potential of curcumin as an antiviral agent by downregulating cccDNA-bound histone acetylation, thereby inhibiting HBV gene replication [18].

Very recently, it has been shown that phytosomal curcumin with higher bioavailability blocks the formation of HCC, improves hepatic histopathology, reduces lipid accumulation and leukocyte infiltration, and diminishes total tumor volume in transgenic mice. Thus, there is a chance of HCC chemoprevention in chronic HBV infection [19].

With a maximum light absorption at 420 nm [20], the cytotoxicity of curcumin as photosensitizer could be expanded by the intravenous blue laser blood irradiation (IVBLBI) [17].

It has been shown in several scientific studies that the blue part of the visible spectrum (400–500 nm) is responsible for the suppression of various pathogens [21,22,23,24].

One hypothesis would be an overload of harmful reducing equivalents throughout the respiratory chain complexes and an extra production of reactive oxygen species (ROS) under blue light irradiation [21].

Several studies have shown that ultrashort laser pulses are capable of inactivating different viruses [25,26,27,28,29].

The mechanism of action initially proposed was that laser radiation produces high frequency resonance vibrations, with a bandwidth of 420–430 nm, capable of destroying the virus capsid by nonthermal treatments, within a few hours [30].

Kingsley et al. proved that the action of the low-power blue laser generates singlet oxygen and, thus, inactivation of viruses occurs through a photochemical mechanism [31].

It is widely known that photobiomodulation is a highly effective method for treating patients with various diseases. Low-intensity (low-energy) laser light used today is safe and may even increase the effectiveness of biological agents in some unresponsive cases [32]. It does not have teratogenic, mutagenic, or carcinogenic properties [33] but, on the contrary, it ensures the protection of organisms against the most different pathogenic factors of biological, chemical, or physical nature, triggering the processes of repair and regeneration inside the living cells [17].

Laser irradiation enhances chromatin recovering, or damaged DNA repair; the biosynthesis of the antioxidant system; the initiation of repairing processes of enzymes (these begin to form, as well as various types of energy-rich substances begin to synthesize); the synthesis of phospholipids, forming cell membranes; the process of reparative regeneration; the proliferation of cellular systems; microcirculation; the biosynthesis of neurotransmitters; the sympathetic activity of the autonomic nervous system; the speed of nerve impulses and the intracardiac conduction; and the neurosecretion process [33,34].

How to kill a virus with laser? Blue laser blood illumination should be preferably used for the correction of immune disorders of various etiologies and as an antimicrobial therapy. For example, in a recently published article by Zhu et al. it was proven by ex vivo and in vivo studies that antimicrobial blue light is a potential alternative or adjunctive therapeutic for infectious keratitis [35]. The mechanism of action of antimicrobial blue light is still not fully understood. A common hypothesis is that antimicrobial blue light excites the naturally occurring endogenous porphyrins or/and flavins in microbial cells and subsequently leads to the production of cytotoxic reactive oxygen species (ROS) [36]. Host–virus interactions in recent literature include the vital role played by host metabolism on viral replication and the proactive participation of mitochondria in this process. Different viruses use distinctive strategies to modulate mitochondrial bioenergetics and enhance viral replication. As a result, energy-yielding metabolic pathways are programmed to provide both energy and biosynthetic resources to drive viral protein synthesis and produce infectious particles. Therefore, metabolic antagonists may prove important not only to outline efficient therapy strategies but also to shed light on the pathogenesis of viral infections [37].

All pathophysiological mechanisms involved in deteriorating cellular function are also not clarified in detail. But it is confirmed that viruses are modulators of the mitochondrial functions, promoting changes in the mitochondrial bioenergetics and triggering an unbalanced mitochondrial metabolism. As a result, the alterations will be both ultrastructural (mitochondria swelling and other morphological changes typical for the apoptotic processes), as well as energetic: A decrease in ATP content and in the energy charge of virus-infected cells. These alterations in mitochondrial physiology and energy homeostasis will proceed cell death due to metabolic stress and the mitochondrial dysfunction and alterations in maintaining the cellular ATP balance.

In our studies, low-level laser therapy allows the consideration of photobiomodulation as an active therapeutic factor, potentiating the curcumin effect [17]. Curcumin features many of the attributes of an ideal photosensitizer for photokilling of pathogens: It is small, can form singlet oxygen in an aprotic environment, and features excellent biocompatibility [38]. UltraCur, a curcumin–whey complex with a 15,000-fold bioavailability and patented in the USA, has a bioavailability equal to 240,000 mg standard curcumin and could be successfully used in connection with the blue laser for treating chronic infectious and age-related diseases [17].

In vitro studies have shown that curcumin can decrease monocyte chemoattractant/chemotactic protein-1 (MCP-1) production in various cell lines. Animal studies have also revealed that curcumin can attenuate MCP-1 expression and improve a range of inflammatory diseases through multiple molecular targets and mechanisms of action. MCP-1, a member of the CC chemokine family, is one of the key chemokines that regulate migration and tissue infiltration of monocytes/macrophages. Its role in the pathophysiology of several inflammatory diseases has been widely recognized, thus making MCP-1 a possible target for anti-inflammatory treatments. However, there is limited data from human clinical trials showing the decreasing effect of curcumin on MCP-1 concentrations and improvement of the course of inflammatory diseases [39].

Curcumin can also downregulate the expression of various proinflammatory cytokines including TNF, IL-1, IL-2, IL-6, IL-8, IL-12, and chemokines, most likely through inactivation of the transcription factor NF-kappa B (nuclear factor kappa-light-chain-enhancer of activated B cells). Together, these findings warrant further consideration of curcumin as a therapy for immune disorders [40]. In the last years, also other natural products, such as hypericin, riboflavin, etc., have been widely investigated as new photosensitizers [17].

Experimental and clinical studies have demonstrated a comprehensive range of remarkable biological effects (antiviral, antimicrobial, immunomodulatory, anti-inflammatory, antioxidant, antiproliferative, antiangiogenic, pro-apoptotic, and hepatoprotective) of curcumin on various cellular and molecular mechanisms (Table 1).

## 4. Curcumin and the Preventive Role in Hepatocellular Carcinoma

In 2015, approximately 257 million people worldwide were chronic carriers of the hepatitis B virus, at risk for severe complications and liver cancer [76,77].

Chronic liver infection with HBV may eventually lead to liver cirrhosis and hepatocellular carcinoma [78], which are major complications and leading causes of death worldwide [79,80].

From over 350 million people infected with HBV worldwide, approximately 600,000 people die from chronic end-stage HBV hepatitis and hepatocellular carcinoma; currently, HBV hepatitis is a major public health problem [81,82].

The International Agency for Research on Cancer reported in 2018 more than 360,000 new cases of hepatocellular carcinoma secondary to HBV infection and about 160,000 new cases after hepatitis C virus infection, representing 76% of all etiologies of this form of cancer [76,83].

Hepatocellular carcinoma (HCC), estimated as the sixth most common cancer in the world, is a complex malignancy, insidiously installed and diagnosed late, with a severe prognosis and whose incidence has doubled, becoming the third leading cause of death from cancer worldwide [76,84,85,86].

Since the early 1980s, a comprehensive HBV immunization program has been introduced as a global preventive measure; World Health Organization is currently supporting this program and has proposed that HBV and HCV infections be eradicated by 2030 [76].

Although modern, direct-acting antiviral drugs have brought a substantial benefit in the therapy of HCV infection with very good rates (>90%) of sustained virologic response and high safety in the advanced stages of the disease, these agents have a disadvantage because they cannot be used extensively for the reason that they have high prices for some patients in the world’s poor countries, and, on the other hand, there are studies that report the recurrence of the disease and the risk of HCC [79].

The main goal of chronic hepatitis B therapy is to reduce viral replication and stop disease progression and local and systemic complications. However, to date, HBV infection has not yet been eradicated because current drug therapies fail to remove covalently closed circular DNA from the patient’s host cells [87].

In case of chronic HBV infection, the drug Lamivudine was first introduced in the therapy of this pathology as an inhibitor of hepatitis B virus reverse transcriptase HBV RT. Lamivudine works as an anti-polymerase/RT activity by inhibiting HBV DNA elongation and, after a few weeks of treatment, reduces by 34 logs of HBV plasma DNA, decreases disease activity, and reshapes the histological structure of the liver [88,89].

Long-term use of Lamivudine frequently causes viral resistance with the possibility of virus replication. The incidence of Lamivudine resistance was reported in 14% to 32% after the first 12 months of treatment, over 38% after 24 months of treatment, and between 53% and 76% after 36 months of initiation of therapy [90].

In recent decades, more information has been obtained on the life cycle of HBV, genetic variability, and pathogenesis. Sequencing of the HBV polymerase (Pol) gene allowed the identification of mutants that occur in the coding of viral polymerase, surface antigen, nucleus, and protein X. The effect of these mutations is also that of resistance to current drugs. The emergence of resistance to antiviral drugs used, poor efficacy in some cases, high costs, and side effects have led to research into herbal antiviral products in the treatment of chronic liver infection and complications such as cirrhosis and hepatocellular carcinoma [77,90,91,92,93,94,95].

Turmeric is the flowering plant *Curcuma longa* of the ginger family, Zingiberaceae. Curcumin (bis-α, β-unsaturated β-dietone) is a polyphenol extracted from the plant *Curcuma longa* that has proven its chemopreventive capacity in carcinogenesis by targeting signaling pathways, regulating the expression of cell adhesion and inhibition of cell proliferation, migration, invasion, angiogenesis, and tumor metastases [96,97,98,99,100].

Curcumin is known as a powerful oxidant, which can stabilize the systems of anti-oxidant enzymes of the liver, reduce the level of superoxide anions (O^2−^), hydroxyl radicals (OH^−^), and increase the level of antioxidants by activating superoxide dismutase (SOD), glutathione peroxidase (GPx), and glutathione S-transferase (GST) [101,102].

Through its polyphenolic action, curcumin works competitively with an inhibitory role on the two isoforms of the enzyme inosine monophosphate dehydrogenase (IMPDH), which is a regulator of intracellular guanine nucleotides. It also plays a particularly important role in signal transduction, energy transfer, glycoprotein synthesis, DNA, RNA, and other multiple processes for cell vitality and proliferation [103,104].

Experimental research of the aqueous extract of *Curcuma longa* rhizome used against HepG 2.2.15 cells containing HBV in their genome showed the suppression of HBsAg secretion from liver cells and mRNA production, without cytotoxic effects. At the same time, a suppression of HBV viral replication was found by transactivating and transcribing the p53 gene (p53 gene like the Rb gene, is a tumor suppressor gene) with increasing p53 protein production [105].

The chemopreventive effect of phytosomal curcumin in the occurrence of HCC in hepatitis B virus was also demonstrated by Teng et al. on a transgenic mouse model [19].

The study started from the fact that in the pathogenesis and progression of HCC was known the role of two viral oncoproteins HBV, hepatitis B X protein (HBx), and pre-S2 as surface mutants [106,107].

It will take a long period, of at least 10–30 years, from the onset of HBV infection to the diagnosis of HCC. Numerous mutations occur throughout the HBV genome. HBx protein mutants on the HBV surface are known to interfere with the processes of stimulation, activation of signaling pathways in liver cells, DNA repair, cell metabolism, proliferation, and apoptosis [108,109].

The pre-S2 protein, another surface mutant, accumulates in the hepatocyte endoplasmic reticulum (ER), mediates the activation of several oxidative stress-dependent pathways, and induces genomic instability and hepatocyte proliferation, involving NF-κB and p38 to regulate cyclooxygenase-2 (COX-2) and vascular endothelial growth factor A (VEGF-A). Teng et al. [19] claim that transgenic mice that have the two HBV oncoproteins can be selected as an experimental model in the production of drugs for preventive and therapeutic purposes in hepatocarcinogenesis.

Its properties as an anti-inflammatory agent, along with its strong antioxidant capacity, have increased interest in curcumin as a therapy for chronic liver disease. Several studies report that curcumin is a potent anti-inflammatory agent and can modulate nuclear factor kappa beta (NF-κB); inhibit phospholipases, cyclooxygenase-2 (COX-2), and 5-lipoxygenase (5-LOX); and decrease the concentrations of TNF alpha, IL-1, IL-6, and agonist of peroxisome proliferator-activated receptor gamma (PPARγ), which has an important role in inhibiting pro-inflammatory pathways [110,111,112,113].

Phytosomal curcumin has been shown experimentally to have effects on peroxisome proliferator-activated receptor gamma (PPARγ) anti-inflammatory activation, pro-inflammatory NF-κB inhibition, suppression of mammalian target of rapamycin (mTOR) deactivation oncogene, improvement of hepatic histopathology (steatosis and necroinflammation), and reduction in volume HCC [106].

The chemoprotective role in HCC would be achieved through the following cellular processes: PPARγ will be produced at the beginning, then heterodimers will form between PPARγ and the retinoid X receptor, which will attract coactivators and bind to the peroxisome proliferator response structures and, thus, regulate the genes of lipid metabolism, anti-inflammatory, and anti-cell proliferative function [114].

Activated PPARy inhibits NF-κB activation, which will induce suppression of NF-B-mediated pro-inflammatory cytokine transcription [115,116] and will inhibit mTOR-mediated highway oncogenic signal activation (mammalian target of rapamycin or sometimes also called mechanistic target of rapamycin) [117,118].

These aspects recommend phytosomal curcumin to be considered as a promising alternative in the long-term treatment, with a chemopreventive role in the occurrence of HCC in patients with chronic HBV infection.

An interesting observation was revealed by epidemiological studies conducted in India, which showed the reduced incidence of digestive cancer through the consumption of *Curcuma longa*, which has proven antioxidant and chemopreventive properties [119].

A recent study focused on the role of hepatic stellate cells (HSC) in the emergence and progress of HCC and the possible protective effect of curcumin. HSCs have a special role in the secretion of soluble factors, such as IL-6, vascular endothelial growth factor (VEGF), and stromal cell-derived factor 1 (SDF-1), and in increasing the level of reactive oxygen species (ROS) to modulate expression of the hypoxia-inducible factor 1-alpha (HIF-1α) in promoting the process of angiogenesis, epithelial-mesenchymal transition (EMT), HCC invasion, and progression. The study data show that curcumin induces protection in the appearance of HCC by purifying ROS and glutathione (GSH), thus blocking HIF-1 and suppressing the expression of connective tissue growth factor (CTGF). The results are promising for the therapeutic effect of curcumin in HCC [120].

In an experimental study to induce the occurrence of HCC by diethylnitrosamine (DEN) in mice, the association among curcumin, leflunomide, and perindopril related to various angiogenic pathways was used as chemoprevention therapy; thus, synergistic inhibition of angiogenesis and prevention of HCC development was achieved [121].

Another major problem in the success of HBV eradication therapy in infected individuals is the persistence of cccDNA residue (covalently closed circular DNA). It is already known from the mode of action of HBV that after the virus invades the cell and rcDNA (partially double-stranded relaxed circular DNA) is left free in the cell nucleus and then converted into covalently closed circular DNA (cccDNA), which is actually a mini chromosome that prints a histonic link. So, HBV infection becomes persistent and recurrent, because this cccDNA structure will serve as a template for mRNA synthesis and viral replication [122,123].

More and more therapeutic protocols with various drugs (nucleoside analogues, disubstituted sulfonamide compounds, cytidine deaminases, zinc-finger nucleases) manage to inhibit HBV replication, but do not rid the cell of cccDNA residue and, worse, recent therapies can target human homologous sequences [124,125,126,127,128,129].

Curcumin may inhibit HBV activity by decreasing the role of the peroxisome proliferator-activated receptor-γ coactivator (PGC)-1α [15] or otherwise, via activating transcription and growth of the p53 [105].

Another hypothesis is that curcumin inhibitory activity in HBV infection is achieved by inhibiting the function of histone acetyltransferase p300 (multifunctional transcriptional coactivator that interacts with numerous transcription factors and protein/histone acetyltransferase activity) that will lead to histone deacetylation related to cccDNA [130,131].

This hypothesis was the basis of an experimental study on a HepG2.2.15 cell line stably transfected with HBV and curcumin treated, after which the ELISA technique assessed the expression levels of HBV surface antigen HBsAg, and HBeAg antigen. Depending on the dose and time, curcumin decreased the expression of HBsAg and HBeAg and played a significant role in remarkably reducing the replication of the intracellular HBV DNA intermediates and HBV cccDNA. In the end, the study proved that curcumin would inhibit HBV gene replication by reducing cccDNA-bound histone acetylation processes and would have a cccDNA-targeted antiviral agent perspective in chronic HBV infection [18].

Curcumin, the polyphenolic compound [132] with antioxidant, anti-inflammatory, antiviral, and chemoprotective properties alone or in combination with other agents or even with the blue laser [133], could be an effective drug for therapy and cancer prevention [134].

## 5. Photodynamic Therapy with Blue Laser and Curcumin in Hepatic Viral Infections

Although the therapeutic properties of light have been known for thousands of years, only in the last two centuries the photodynamic therapy (PDT) has been developed.

PDT is a modern treatment that uses light (phototherapy) together with a light-sensitive chemical that, in the presence of molecular oxygen, causes cell death in the target tissue by phototoxicity.

Anti-infectious, light-mediated effect was rediscovered by chance in 1900: *Paramecium caudatum* incubated with acridine died when exposed to certain wavelengths, but only in the presence of oxygen [135].

One year later, Niels Finsen ingeniously used light therapy for smallpox and skin tuberculosis, and in 1903 won the Nobel Prize for his phototherapy work [136] “in recognition of his contribution to the treatment of diseases, especially lupus vulgaris, with concentrated light radiation, whereby he has opened a new avenue for medical science” [137].

The term “photodynamic” was for the first time used in 1907 by Herman von Tappeiner, who defined the photodynamic action [138].

Since then, PDT became a well-studied therapy for infections, nonmalignant diseases [139], and in some countries has become a standardized protocol alongside radiotherapy and chemotherapy in anticancer treatments.

The purpose of PDT is to induce various cellular responses, from the inactivation of bacteria and viruses to the necrosis or apoptosis of pathological cells, using the three absolutely necessary elements: Laser light, molecular oxygen, and a nontoxic, light-sensitive substance, named photosensitizer (PS).

The first photosensitizer, called “hematoporphyrin derivative” (HpD) or, later (when purified), “Photofrin”, was invented by Dr. Thomas Dougherty and colleagues in 1970 at Roswell Park’s Department of Experimental Biology, USA, from a mixture of water-soluble porphyrins. With this product he began to successfully treat cancer with PDT in preclinical models, and in 1975 he treated the first cases of skin cancer. In 1978, Dr. Dougherty carried out the first human clinical trial with control group [136].

It turned out that the method acted not only on the tumor, but also destroyed the blood supply of the remaining cancer cells around the tumor, preventing their proliferation and expansion. After treatment, the procedure induces inflammatory and immune responses and research has shown that the extensive action on the immune system makes it able to target and eliminate other cancer cells from the body [140].

Photosensitizers (PS) are pure chemicals with a well-specified composition, which, when introduced into the body, will preferentially accumulate in the target tissue and, after irradiation with a certain wavelength in the visible range, absorb it strongly and pass through a singlet state, followed by a long-lasting excited triplet state, during which cytotoxic reactive oxygen species (ROS) are generated through various intrinsic mechanisms, which will interact with pathogenic agents and malignant cells and tissues, which they will destroy.

Although it is still widely used, Photofrin, as a photosensitizer from first generation, has disadvantages including a relatively low absorption maximum at 630 nm (so, difficult to use for large malignancies, where light penetration will be more difficult throughout the whole volume) and, for the patients, the residual photosensitivity of the skin over several weeks or even months can bother and irritate them greatly [136,141].

PDT has been extensively investigated and applied in the last decades, proving to be an effective method of treatment. As a two-step procedure, PDT begins with the administration of the photosensitizer, followed by exposure to light, in order to obliterate the pathogens or the malignant structures [142].

Subsequently, acute inflammatory processes trigger warning signals to the adaptive arm of innate immune system for generating the specific cytotoxic T lymphocytes that have the capability to directly identify and eliminate the self-altered cells and release even more mediators.

This effect of the adaptive immune response has highlighted in recent years the great value of PDT for long-term control and immune memory to stop recurrences [143].

Due to all these aspects and proving to be a minimally invasive treatment modality, the intense interest for PDT has increased exponentially. The global efficacy of PDT overlies the characteristics of activation laser light and in situ dosimetry (selection of PS, wavelength of light, and layout of the target tissue and its small-scale environment) [144].

When put into action appropriately, PDT has no long-term undesirable secondary effects, can be applied with maximum accuracy, is not as invasive as other treatment modalities, can be repeated many times at the same site if needed, is most often less expensive, and can be done as outpatient therapy [145]. However, with all its proven success, PDT is still underrated and not so widely used [142].

Also, interstitial photodynamic therapy (I-PDT) is showing signs of future success for advanced well-localized cancers but can be encountered only in few clinics [146].

Cancer stem cell model opened new plans of action designed to achieve the major aims: Efficient treatment and cancer’s prophylaxis, targeting signaling pathways such as Wnt, Notch, and Hedgehog (Hh) with natural compounds, for example, curcumin, in order to minimize the risk of relapses [147].

Due to the fact that curcumin is a potent inhibitor of NF-κB signaling and of the expression of viral oncogenes E6 and E7, in decreasing the integrase and viral protease of HIV-1, in the intracellular accumulation of p53, and, consequently, in decreasing the RNA level of intracellular HBV, several studies have been performed in an attempt to use it as a photosensitizer in photodynamic therapy [148].

PDT as a state-of-the-art medical technology, using blue light (400–470 nm), can effectively fight bacterial, fungal, and viral infections.

Photoreceptors, which are specific cellular structures, can absorb only specific wavelengths of light.

Light interaction with these photoreceptors will trigger a cascading cellular signal, which will affect the chemical behavior, the metabolism, the movement, and the expression of genes inside the cell. All associated enzymes and/or proteins will be affected [133].

An example for the absorption of different wavelengths inside cells is the process in the mitochondrial respiratory chain, where complex 1 (NADH dehydrogenase) absorbs blue and ultraviolet light [133].

Blue light releases nitric oxide (NO), known to be a growth, immune, and neuro-modulator, as well as a stimulator of stem cell proliferation, playing critical roles in pain reduction, vasodilation, and angiogenesis through c-GMP (cyclic guanosine monophosphate) pathway.

The blue laser has a strong anti-inflammatory effect by reducing proinflammatory cytokines (for example, IL2, IL6, TNF-alpha) and acts on cellular signaling pathways (e.g., NF-kB) and other factors that contribute to a variety of diseases (CRP, leptin, chemokines, etc.).

Blue light is effective in treating infections by producing ROS (especially in combination with photosensitive substances such as riboflavin or curcumin) [17,133].

Great efforts are currently being made to explore the clinical value of blue light in the fight against viral infections, an example being the current COVID-19 pandemic [149].

The important bactericidal effect of blue light spectrum when acting on most microbes has been demonstrated; pulsed wave blue light is more efficient than continuous wave [150].

In addition to blue light laser devices, updated technology has designed and manufactured LED devices that are much cheaper and easier to use by patients to fight viral infections, even at home [149,150].

The review of scientific papers published so far shows that the antibacterial effect is caused by the action of blue light that activates endogenous bacterial chromophores and photosensitive receptors that release reactive oxygen species (ROS) capable of triggering cellular apoptosis.

Another possible mechanism would be the action of blue light on the integrity of the cell membrane, with the decrease of the transmembrane potential and, consequently, the rapid disturbance of cellular functions [151].

The latest possible mechanisms by which blue light would annihilate bacteria, currently proposed but requiring further scientific studies to be confirmed, refer to the modification of A-DNA and the upregulation of the prophage genes [149,152,153].

PDT with blue light in the 420–430 nm wavelength band (the maximum absorption of curcumin) applied to blood plasma inactivates herpes simplex virus and human immunodeficiency virus, and prolonged exposure to blue light inactivates leukemia virus (in murine) [154,155,156,157].

Particular problems raised by viral infections are those related to current ineffective drugs for many viral diseases, the emergence of resistance, sometimes formidable side effects, and high costs, which have led specialists to discover new compounds or effective antiviral treatment [103].

Due to the 15 different polyphenols it contains, curcumin has an exceptional antiviral activity, competing with inosine monophosphate dehydrogenase (IMPDH), an enzyme that limits starting just from the beginning the synthesis of guanine nucleotides and which activates signaling pathways in the evolution of viral infections to neoplasms [158].

The antitumor effects of curcumin are due not only to its action on various signaling pathways, but also on potentiating the efficacy of the immune system for removing the tumor cells just from the beginning of the proliferative processes, suppressing the neoplasm development [159].

Bose et al. highlighted the immunomodulatory role of curcumin by affecting the ratio of CD4 (+) T cells/CD8 (+) T cells, with the decrease of Treg cells and the suppression of T cell apoptosis restoring the immune vigilance against tumor cells and, consequently, the retrogression of the tumor [160].

At the initiation of the tumor process, naïve T cells have a special role because they are becoming operative, set into motion the microenvironment of the tumor formation, and fight to remove immunogenic neoplastic cells [161].

Cytotoxic T lymphocytes (CTL) arise from the differentiation of CD8+ T cells, which play the leading role as antitumor cells; meanwhile, CD4+ T helper1 (Th-1) will support the T cell switch on, increase the CTL cytotoxicity, and can foster the development of the antineoplastic task of the macrophages and NK cells, generating different pro-inflammatory cytokines [162,163].

Curcumin could transform regulatory T cells into helper T cells (Th-1), with antitumor results [159].

The anticancer activity of curcumin by targeting and modifying the regulatory T cells into T helper ones, could be enhanced by the blue laser photobiomodulation so that HBV-infected hepatocytes are more easily eliminated.

Because curcumin proved to be a very good photosensitizer used also in the food industry, Randazzo W. et al. [164] measured the antiviral activity of blue light-photoactivated curcumin on surrogate norovirus, feline calicivirus (FCV), and murine norovirus (MNV).

PDT with LEDs’ blue light-activating curcumin with 3 J/cm^2^ against MNV-1 distorted the viral capsid and reduced FCV titers, so that photoactivated curcumin could be used in food security to reduce viral contamination [164].

Even without being illuminated, curcumin has a very good effect on the microvessels in the circulatory system, impact that is maximized by lighting, resulting in a faster decrease of an important number of vessels and their diameter under the action of light and curcumin, with important applications in the vascular pathology and the control of blood supply in case of tumors [165].

## 6. Conclusions

Blue laser light and UltraBioavailable Curcumin could be a new, valuable alternative for medical applications in chronic B viral hepatitis and hepatocellular carcinoma, saving millions of lives.

## Figures and Tables

**Table 1:** Molecular targets and protective effects of curcumin on pathogenic pathways [41,42,43,44,45,46,47,48,49,50,51,52,53,54,55,56,57,58,59,60,61,62,63,64,65,66,67,68,69,70,71,72,73,74,75]. ▼ = “decrease”, ▲ = “increase”.

Biological Effects	References	Molecular Targets/Pathogenic Pathways	Conclusions
**Immunomodulator**	Yang, et al. [41]	Reduces the receptor activator of nuclear factor-kappa-B ligand (RANKL) ▼ Akt/NF-κB/NFATc1 pathways ▼	Curcumin ameliorated the RANKL-mediated differentiation, fusion and maturation of osteoclasts and had an immunomodulatory effect on macrophage polarization; the protective effect of curcumin on osteoclast genesis was mediated by attenuating the up-regulation of Akt and p65 phosphorylation and the activation of the downstream transcription factor NFATc1 (Nuclear Factor of Activated T-cells, cytoplasmic 1).
	Wu, et al. [42]	NF-κB, MAPK, Akt and pBAD pathways ▼	Curcumin treatment reduced activation of the NF-κB, MAPK (mitogen-activated protein kinase), Akt and pBAD (systematically araBp, a promoter found in bacteria) pathways either systemically, or within the inflamed kidneys. These findings suggest that natural food supplements could become an alternative approach to ameliorating immune-mediated kidney diseases.
	Tuyaerts, et al. [43]	COX-2 expression in B cells, NK and T cells. Myeloid -Derived Suppressor Cell (MDSC): CD15^+^ & CD33^+^ granulocytic, and CD33^+^ monocytic. Expression of HLA-DR/CD45^+^ leukocytes	It is observed a downregulation of MHC expression by leukocytes, a reduction in the frequency of monocytes and a decreased inducible co-stimulator (ICOS) expression by CD8^+^ T cells upon Curcumin Phytosome (CP) intake, while the level of CD69 on CD16- NK cells was upregulated; did not find significant changes in inflammatory biomarker levels, frequencies of other immune cell types, T cell activation and COX-2 expression.
	Mollazadeh, et al. [44]	Dendritic cells, macrophages, mast cells, natural killer cells, eosinophils, neutrophils, B cells, CD8+ T cells, TH1, TH2, TH17 & regulatory T cells	Curcumin is a natural anti-inflammatory compound able to induce the expression and production of IL-10 and enhancing its action on many tissues.
	Boroumand, et al. [45]	Neutrophils, macrophages, monocytes, NK cells, dendritic cells (DCs), T and B cells Down regulating the expression of growth and survival promoting genes including c-Myc, BCL-XL and NF-κB ▼	This review discusses current knowledge on the immunomodulatory, anti-inflammatory and antioxidant roles of curcumin, with the hope of recruiting curcumin as a therapeutic agent in the future therapeutic regimen.
	Bai, et al. [46]	Inhibition of nuclear factor-kappa B (NF-κB) ▼	Curcumin is a potent inducer of apoptosis—an effector mechanism used by macrophages to kill intracellular *Mycobacterium tuberculosis (MTB)*. Curcumin enhanced the clearance of MTB in differentiated THP-1 human monocyte and in primary human alveolar macrophages. Curcumin was an inducer of caspase-3-dependent apoptosis and autophagy.
	Antiga, et al. [47]	Th22 cells, a subpopulation of T cells ▼	In conclusion, curcumin was demonstrated to be effective as an adjuvant therapy for the treatment of psoriasis vulgaris and to significantly reduce serum levels of IL-22.
	Castro, et al. [48]	NF-kB, iNOS IL-6, TNF-alfa, IL-1 beta ▼	Curcumin modulates the T lymphocyte response impairing proliferation and interferon (IFN)-γ production through modulation of T-box expressed in T cells (T-bet), a key transcription factor for proinflammatory T helper type 1 (Th1) lymphocyte differentiation, both at the transcriptional and translational levels. Reduces nuclear factor (NF)-κB activation in T cell receptor (TCR)-stimulated NOD lymphocytes and secretion of proinflammatory cytokines and nitric oxide (NO).
**Anti-inflammatory**	Lee, et al. [49]	IFN-γ, TNF alfa, IL1 beta, 6, 10, 13. 17 ▼ NF-κB/COX-2 pathway and iNOS ▼	Authors conclude that *C. longa* and *A. hookeri* co-treatment synergistically inhibit inflammation by regulating the NF-κB/COX-2/iNOS pathway.
	Li, et al. [50]	Macrophage (M); Lipopolysaccharide (LPS), IFN-γ promote M1-type macrophage; IL-4, 10 and IL-13 promote M2-type macrophage polarization	Macrophages play a pivotal role in non-alcoholic fatty liver disease (NASH) development. Evidence for natural products or their active ingredients in the modulation of macrophage activation, recruitment, and polarization, as well as the metabolic status of macrophages were assessed.
	Almatroodi, et al. [51]	TNF-α, IL-6 ▼ p53 expression▼ Glutathione peroxidase (GPx), superoxide dismutase (SOD), catalase (CAT) Total Antioxidant Capacity (TAC)%▲	Co-administration of curcumin (50 mg/kg) with BaP (50 mg/kg) exhibited significant reduction in TNF-α and the IL-6 level; significantly decreased the number of TUNEL-positive cells. Curcumin plus BaP decreased the p53 protein expression as compared with the BaP group and intensity of positivity was low as compared to the BaP only treated group. In addition, curcumin administered to BaP induced rats confirmed the improvement in SOD, CAT and GPx, total antioxidant capacity, and oxidative stress biomarker; significantly decreased the accumulation of cells in the G2/M phase and reduced the apoptotic cell death level increased by BaP.
	Vitali, et al. [52]	IL-6, TNF-α and MCP-1 transcription factor NF-κB ▼	Results show that delivery of curcumin nanoparticles directly into the vaginal tract of mice infected with HSV-2 abrogated CpG-oligodeoxynucleotide (ODN) induced inflammatory vaginal pathology and diminished the production of pro-inflammatory cytokines IL-6, TNF-α and MCP-1 in vaginal and cervical tissues.
	Ullah, et al. [53]	Cycloxygenase-2 (COX-2)▼ Inhibitory kappa B Kinase (IKK) and TANK binding kinase-1 (TBK-1) of Toll Like Receptor 4 (TLR4) pathway ▲	A continuous computerized examination of 14 compounds comprising curcumin, its derivatives and analogues were investigated as inhibitors of signaling proteins: cyclooxygenase-2 (COX-2), Kappaβ kinase inhibitor (IKK) and TANK-1 binding kinase (TBK-1), and Toll Path Like Receptor 4 (TLR4) involved in inflammation. This study recommends 6-Gingerol, Yakuchinone A and Yakuchinone B as the best inhibitors of COX-2, IKK and TBK-1 respectively among the selected curcumin analogues, which could be considered as potential anti-inflammatory agents in the search for new medication against inflammation.
	Shimizu, et al. [54]	NF-kappa B TNF-alpha, IL-1beta myeloid differentiation protein 2-Toll-like receptor 4 co-receptor pathways▼ activates peroxisome proliferator-activated receptor-gamma (PPAR-gamma) ▲	A number of studies have reported the efficacy of curcumin and the mechanisms by which its anti-inflammatory activity could treat various lifestyle-related conditions associated with chronic inflammation, including atherosclerosis, heart failure, obesity, diabetes and other related diseases, such as dementia. Most of these studies have involved animal experiments; however, there are several reports on the benefits of curcumin use in humans.
	Szebeni, et al. [55]	human PBMCs (peripheral blood mononuclear cells) NF-kB; TNF-alfa, IL4, IL6 ▼	The effects of treatment with curcumin analogues in the inflamed colon of rats demonstrated a significant reduction in total tissue myeloperoxidase (MPO) activity, like the biological agent infliximab. Mannich curcuminoids reported herein possess a powerful anti-inflammatory activity.
	Wal, et al. [56]	NF-κB, TNF-α▼ COX-2, inducible nitric oxide synthase (INS) and lipoxygenase (COX). INOs, LOX ▼	Curcumin has the potential for curing inflammatory diseases as it blocks the mechanism of reactive oxygen species generation via inhibiting oxidative stress. Curcumin has prominent effect on inflammatory mediators like cytokines as a result of which it blocks the oxidation process in mitochondria of the cell and reduces inflammation.
	Chen, et al. [57]	TNF-α, IL-1β and IL-6 as pro-inflammatory cytokines▼ NF-κB p65 ▼	After four weeks of administration of combined two natural products with curcumin resulted in a decrease in the inflammatory cell infiltration, less thickness of the synovium and less volumetric increase in the synovial space at collagen-induced rat model arthritis; the level of TNF-α, IL-1β and IL-6 was significantly decreased in serum. Curcumin combination was able to inhibit the expression of NF-κB p65 and TNF-α which were closely related to the inflammatory process.
	Panahi, et al. [58]	TNF-a, IL-6, TGF-b and MCP-1▼ macrophages monocytes NF-κBp65▼	Study suggests a significant decrease in serum concentrations of TNF-a, IL-6, TGF-b and chemoattractant protein-1 (MCP-1) cytokines following curcumin supplementation in subjects with metabolic syndrome.
**Antioxidant**	Paciello, et al. [59]	Nuclear factor (erythroid-derived 2)-like 2 ») or Nrf2/Heme oxygenase-1 (HO-1) pathway; ROS downregulating p53 phosphorylation ▼ NF-κB, STAT-3 ▼	The authors used two different polyphenols in adjuvant chemotherapy in several malignancies to avoid the chemoresistance and ototoxicity of the drug cisplatin. Both polyphenols had an antioxidant and self-protective action by up-regulating the Nrf-2/HO-1 pathways and down-regulating p53 phosphorylation; but only curcumin has been able to influence the inflammatory pathways that counteract NF-κB activation. In human cancer cells, curcumin transforms the antioxidant effect into a pro-oxidant and anti-inflammatory one. Curcumin has permissive and chemosensitive properties by targeting chemoresistant factors of cisplatin phosphorylation of Nrf-2, NF-κB and STAT-3. Therefore, polyphenols, mainly curcumin, which target ROS modulated pathways can be a promising tool for the treatment of cancer. Due to their biphasic antioxidant activity in normal cells under stress and pro-oxidant in cancer cells, these polyphenols are likely to involve an interaction between key factors Nrf-2, NF-κB, STAT-3 and p53.
	AlBasher, et al. [60]	(MDA) malondialdehyde, {NO} nitric oxide ▼, {GSH} reduced glutathione, {GPx} glutathione peroxidase, {SOD} superoxide dismutase, {CAT} catalase) ▲ Nrf2 ARE antioxidant response elements	This study was undertaken to assess the effects of resveratrol (RSV) and curcumin (CUR) on oxidative damage induced by insecticide fipronil (FPN). Co-treatment with RSV and CUR, mainly in combination, markedly alleviated the toxic effects and oxidative damage induced by FPN. Thus, liver enzyme activities, renal injury biomarkers, and lipid profiles in the sera, as well as the MDA, NO, and GSH concentrations and GPx, SOD, and CAT activities (liver, kidney, and brain), were normalized. RSV and CUR repaired oxidative disorders through the Nrf2 heterodimer that bound to antioxidant response elements (AER) and regulated the expression of antioxidant enzyme genes such as GPx, SOD and CAT.
	Konak, et al. [61]	SOD, CAT, GPx ▲ ROS, NO▼ total antioxidant capacity (TAC) total oxidant capacity (TOC)▲	Study investigated the antioxidant effect of curcumin, a phytochemical, on the blood tissue of rats. When the TAC and TOC levels of curcumin-supplemented feeding group were examined, the level was higher than the control group (P <0.05). Results of the study show that curcumin strengthens the antioxidant defense system.
	Nawab, et al. [62]	CAT, SOD, GSH-Px and T-AOC▼	The effects of increasing concentrations of dietary curcumin on the antioxidant parameters of layers maintained under high-temperature conditions for nine weeks were evaluated. Laying hens in all curcumin treatment groups had slightly higher activities of CAT, SOD, GSH-Px, and T-AOC in the liver, heart, and lungs, compared with heat stressed control group. It was concluded that dietary curcumin given to laying hens under heat stress may enhance their antioxidant status and alleviate the detrimental effects of stressful environmental conditions.
	Nawab, et al. [63]	IL-6, IL-1β, TNF-α, ▼ TLR4, NF-κB; PCNA (proliferating cell nuclear antigen) ▼	This study aimed to investigate the effect of curcumin supplementation on TLR4 mediated non-specific immune response in liver of laying hens under high-temperature conditions and heat stress. Authors showed that in the curcumin group treatment had reduced inflammatory responses (IL-6, IL-1β, TNF-α) as compared to control group. Furthermore, PCNA, TLR4 and its downstream gene expression as well as protein expression (TLR4, NF-κB and PCNA) were significantly downregulated in heat stress curcumin supplemented group as compared to control.
	Meshkibaf, et al. [64]	Methionine Sulfoxide Reductase A (MSRA) SOD, CAT, GPx ▲	Effect of curcumin on arthritis induced by adjuvant in rats, considering changes in methionine sulfoxide reductase A (MSRA) expression and antioxidant enzymes levels were analyzed. Curcumin can be used against inflammation. The increasing level of MSRA can be due to the antioxidant effect of curcumin. The enzymatic level changes (MSRA, SOD, CAT and GPx) may interfere with the aging process and delay it.
	Borra, et al. [65]	MDA: Malondialdehyde, PC: Protein carbonyls, GSH: Reduced glutathione TAC: Total Antioxidant Capacity ▲; ROS▼	Curcumin has the ability to protect the biomolecules like proteins, lipids and DNA from hydroxyl radical oxidation. These results demonstrate that exposure to OH in serum and plasma is a good experimental model for studying ROS-induced toxicity and evaluating the protective effects of various agents in vitro.
	Jat, et al. [66]	GSH▲ Lipid peroxidation (LPO)▼ Protein carbonyl▼; ROS▼	This study shows the strong anti-oxidative effects of curcumin and mitochondrially targeted curcumin against the lipid peroxidation, protein carbonylation and mitochondrial permeability transition induced by tert-butylhydroperoxide. Both curcumin and mitochondrially targeted curcumin significantly enhanced endogenous reduced glutathione level in the mitochondria thus preserving mitochondrial defense system against oxidative stress.
**Anti-angiogenic**	Norooznezhad, et al. [67]	CD68+ and CD11b+ cells; IL-1, IL-6, TNF-α, VEGF ▲ hypoxia-inducible factor 1α (HIF-1α) ▲	Curcumin has a strong antioxidant potential, can decrease oxidative stress and it is able to inhibit the mentioned inflammatory and angiogenic factors such as IL-1, IL-6, TNF-α, VEGF, MMPs, and HIF-1α.
	Mohapatra, et al. [68]	Glutathione (GSH) ▼ Malondialdehyde (MDA) ▼ Superoxide dismutase (SOD) ▲	The combination of curcumin and ASA (acetylsalicylic acid) significantly reduced foot edema and the formation of carrageenan-induced granuloma. Concomitant use of curcumin with ASA decreased malondialdehyde levels, while relatively increased superoxide dismutase and reduced glutathione, while proving protection against (histologically) induced hepatotoxicity by ASA.
**Pro-Apoptotic/Antiproli-ferative**	Hassan, et al. [69]	p53 level caspase activation ▲ tyrosine-regulated kinases (DYRKs) ▼ 26S proteasome activity ▼	Potent role of curcumin as epigenic modulator, influence on transcriptional factors, antioxidant effects, mediation of inflammatory cytokines and angiogenesis modulation was proved. Curcumin is a potent proteasome inhibitor that increases p53 level and induces apoptosis by mitochondrial caspase activation. Curcumin also disrupts 26S proteasome activity by inhibiting DYRK2 in different cancerous cells, resulting in the inhibition of cell proliferation.
	Rainey, et al. [70]	AMP-activated protein kinase *NRF1* (Nuclear Respiratory Factor 1); Nuclear factor (erythroid-derived 2)- like 2, also known as NFE2L2, Mitochondrial transcription factor A (TFAM) TFEB (Transcription Factor EB) G2/M cell cycle Peroxisome proliferator-activated receptor gamma coactivator 1-alpha (PGC-1α) -related signaling pathway. Activator protein 1 (AP-1), Caspase-10	Multiple in vitro and in vivo preclinical studies have shown that curcumin is effective against various types of cancer. These potent the effects are determined by the ability of curcumin to induce G2/M cell cycle arrest, induction of autophagy, activation of apoptosis, disruption of molecules signaling, inhibition of invasion and metastasis and increasing the effectiveness of current chemotherapeutics. Low levels of curcumin enhance mitochondrial biogenesis in cells and tissues, mainly through the induction of the PGC-1α-related signaling pathway.
	Guo, et al. [71]	Telomerase reverse transcriptase (TERT) ▲; Malondialdehyde (MDA) ▼ glutathione (GSH); superoxide ▲; dismutase (SOD)▲; glutathione peroxidase (GSH-Px); TNF-α and IL-1β in the cerebral homogenates ▼ TERT mRNA expression level▲	Study evaluates the possible anti-apoptotic, antioxidant and anti-inflammatory effects of curcumin on the neurotoxicity caused by Acrylamide (ACR) in rats. Curcumin increased TERT mRNA expression level, suggesting curcumin might exert anti-apoptotic activity in ACR-induced neurotoxicity partly through maintaining TERT-related anti-apoptotic function. Curcumin at the dose of 100 mg/kg significantly decreased the levels MDA and IL-1β and TNF-α; increased levels GSH and SOD.
	Liu, et al. [72]	G2/M arrest via possible inhibition of cell cycle-related proteins including Cyclin-dependent kinase 1 (CDK1), cyclin B1, and “cell division cycle” (CDC25C). Extracellular signal-regulated kinase (ERK1/2) and Protein Kinase B (Akt) pathways. Transforming growth factor-β (TGF-β)/Smad pathway. Notch signaling pathway.	This study shows that curcumin could suppress the proliferation and epithelial-mesenchymal transition (EMT) in lens epithelial cells (LECs) and it might be a potential therapeutic protection against visual loss induced by posterior capsule opacification (PCO).
	Chen, et al. [73]	▼ miR-21 in SU-DHL-8 cells, regulating Von Hippel-Lindau (VHL) expression in diffuse large B-cell lymphoma (DLBCL) cell line.	Curcumin exerts its anti-cancer effects, partly by targeting special microRNAs, in human cancers. MiR-21 is a key oncomir in carcinogenesis of multiple human cancers. Curcumin exerted its anti-proliferation, anti-migration, anti-invasion, and pro-apoptosis functions, at least partly, by repressing miR-21 and regulating VHL expression in DLBCL cell line. Study proved a possible molecular mechanism of curcumin-mediated anti-cancer effect.
	Ma, et al. [74]	Caspase 3 and BAX (protein Bcl-2–associated X) ▲ reducing the expression of Bcl-2 in (human tongue squamous cell carcinoma) CAL 27 cells ▼	This study focused on the mechanism underlying the therapeutic effect of curcumin against tongue cancer (TC). The effects of curcumin at different concentrations on the proliferation, migration, apoptosis, and cell cycle for TC cells were verified in vitro. Study revealed the anti-proliferative and pro-apoptotic roles of curcumin in CAL 27 cells, probably due to its role in modulating oxygen production and metabolism. Curcumin might be explored as a promising therapeutic method to improve TC treatment.
	Muangnoi, et al. [75]	Caspase-3 and -9 activities and expression ▲ Bax and Bcl-2 protein expression LC3-II protein level ▲	The aims of the present study were to determine the anti-proliferative effects and mechanisms of curcumin diethyl disuccinate (CurDD), a succinate ester prodrug of Curcumin (Cur), in vitro, in comparison with Cur. These results indicated that the bioavailable fraction (BF) of CurDD had anti-proliferation effect on HepG2 cells by apoptosis induction in HepG2 cells at significantly higher levels than that of Cur. Incubation of HepG2 cells with the BF of Cur increased caspase-3 and -9 activities. These results indicated that treatment of HepG2 cells with the BF of CurDD increased and, respectively, decreased the expression of Bax and Bcl-2 proteins, to a higher extent than in the case of the BF of Cur. The BF of CurDD increased the level of LC3-II significantly higher than the BF of Cur.

## Abbreviations

AN	Analog nucleotides
cccDNA	Covalently closed circular DNA
GHSS	Global Health Sector Strategy
HCC	Hepatocellular carcinoma
HCV	Hepatitis C virus
HBV	Hepatitis B virus
IFN	Interferon
IVBLBI	Intravenous blue laser blood irradiation
MCP-1	Monocyte chemoattractant/chemotactic protein-1
PBM	Photobiomodulation
PDT	Photodynamic therapy
ROS	Reactive oxygen species
WHO	World Health Organization
